# Typical ECG findings in an unconscious patient

**DOI:** 10.1007/s12471-016-0909-4

**Published:** 2016-10-26

**Authors:** R. Joustra, F. N. Polderman, J. L. Smeets, M. C. Daniëls, M. Boulaksil

**Affiliations:** 1 Department of Cardiology, Jeroen Bosch Hospital, ’s-Hertogenbosch, The Netherlands; 2 Department of Cardiology, Radboud University Medical Center, Nijmegen, The Netherlands; 3 Department of Intensive Care Medicine, Jeroen Bosch Hospital, ’s-Hertogenbosch, The Netherlands

A 62-year-old woman known with a depressive disorder but unremarkable cardiovascular history was admitted to our emergency department in a state of unconsciousness. Her husband had found her unconscious at home and called an ambulance. She had no history of syncope or suicidality. Physical examination demonstrated a Glasgow Coma Scale of E_1_M_5_V_1_ without any lateralising neurological symptoms. She was haemodynamically stable, but was intubated because of failure to maintain airway tone due to unconsciousness. She had no fever. Laboratory results showed: sodium 150 mmol/l, potassium 3.2 mmol/l, a normal glomerular filtration rate, haemoglobin 7.6 mmol/l; normal thrombocytes and leucocytes. The ECG on admission is shown in Fig. 1.

**Fig. 1:**
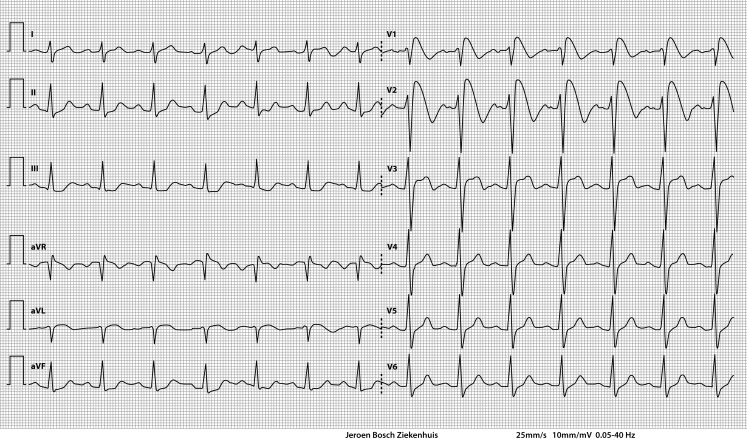
ECG on admission

1. What is your diagnosis?
2. What is the most likely mechanism?

## Answer

You will find the answer elsewhere in this issue.

